# A structured reflective process supports student awareness of employability skills development in a science placement module

**DOI:** 10.1002/2211-5463.13158

**Published:** 2021-05-02

**Authors:** Luciane V. Mello, Tunde Varga‐Atkins, Steve W. Edwards

**Affiliations:** ^1^ School of Life Sciences University of Liverpool UK; ^2^ Centre for Innovation in Education University of Liverpool UK; ^3^ Institute of Infection, Veterinary and Ecological Sciences University of Liverpool UK

**Keywords:** employability, higher education, placements, portfolio, skills reflection, transferable skills

## Abstract

Placements are often an extra‐curricular activity of a science degree. This study reports on the outcomes of a final‐year credit‐bearing 6‐week placement module that was specifically designed to develop and enhance students’ employability skills. A key element of this module was that the student placements were not only evaluated from a science perspective, but also evaluated with an emphasis on meaningful reflection and evaluation of employability skills development. Students recorded their levels of confidence in skills before, during and after the placement via an Online Reflective Log, as part of a module’s summative assessment. The results showed that taking part in the placement and conducting their own independent research helped students to make connections between their scientific knowledge, otherwise constrained within the walls of the undergraduate science laboratory, and the wider impact of their research on society. Another theme that emerged concerned career choices and aspirations, and the placement experiences either confirmed prior choices or opened new horizons. The Online Reflective Log helped students to feel supported by their university supervisor who were at a distance. Feedback on their tasks prompted students to reflect on the scientific and personal skills while being engaged in scientific activities during placement. Students agreed that they had further developed their employability skills during the placement and acknowledged that it was challenging to acquire evidence of skill development. However, students appreciated the usefulness of this reflection in relation to their future career development.

## Graphical abstract

Using an Online Reflective Log as summative assessment during a 6‐week, credit‐bearing placement module, students record their perceptions of skills development and planned activities, supported by their university supervisor. Even during a short placement, this structured and reflective approach has shown to help students achieve better awareness of their skills, connecting their scientific understanding to real‐world research and skills development.

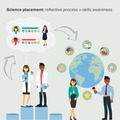

**Abbreviations**
HEIs: Higher education institutions
NSS: National Student Satisfaction
RPR: recording, planning and reflection
TEF: Teaching Excellence and Student Outcomes Framework

In 1997 the Dearing Report [1] identified the need to enhance a range of ‘key’ competencies for university students to enhance their life‐ and employability‐skills. Universities have responded accordingly, by either embedding these skills, including communication, numeracy, IT and learning skills, into core modules, or by designing bespoke modules that develop such skills alongside the academic curriculum [2, 3]. These changes have transformed skills development in students in spite of initial reservations by some staff that the university curriculum should only focus on academic subjects. The development of these skills had greatly aided student learning, but the focus has recently shifted to employability skills and preparedness of students for the workplace [4, 5, 6]. The inclusion of graduate employability statistics in the UK National Student Satisfaction (NSS) survey and the Teaching Excellence and Student Outcomes Framework (TEF) has highlighted the importance of this metric for universities and prompted further efforts to strategically embed employability skills more visibly in curricula [7]. The NSS is an annual survey for final‐year undergraduate students aimed to gather feedback on their experiences during their course; the TEF assesses excellence in learning and teaching and how higher education institutions (HEIs) ensures outcomes for their students in terms of graduate‐level employment or further study.

Employers often report that they are generally satisfied with the academic abilities and standards of students and the value of their degree qualifications, but many still report that students are often not ‘workplace ready’ and find it difficult to transition to a workplace environment from the academic environment [8, 9, 10]. This may be more marked when students seek employment beyond their academic discipline, and less apparent when students, for example, transition to study for a higher degree for which their previous academic experiences adequately prepare them. Work experience can help students prepare for the workplace, and both students and employers value the maturity, experience and understanding of the work environment that students gain during placement years [11, 12, 13]. However, the number of available year‐long placements is declining and the national competition between students for such placements is fierce. Thus, not all students who seek a year‐long placement and who would benefit from the experiences are successful in obtaining these opportunities.

Many students do not fully recognise, reflect on, evidence and evaluate the full sets of skills that they have acquired during their university education, and even if they do, they are often unable to articulate specific examples of how they can demonstrate competency in such skills at interview or in job applications [14]. Students are often assessment‐driven, and unless employability skills are an integral part of the assessment process, they are often seen as optional, extra‐ or co‐curricular, activities. Students may not see the relevance of these skills, or more generally, how their university experiences prepare them for the workplace. However, skills development that is based on critical self‐reflection [15, 16] and experiential learning [17, 18] can lead to greater student awareness and engagement, and so be more effective than the didactic teaching of such skills.

There are several definitions of employability skills, also known as transferable skills. The definition adopted in this study is that of Knight and Yorke [19] as ‘a set of achievements, understandings and personal‐attributes that make individuals more likely to gain employment and be successful in their chosen occupations’. While employment should be regarded as an outcome, employability should be viewed as a lifelong attribute [20]. Lifelong learning involves understanding how to learn, reflect and progress [21]. It is founded on the premise that a greater awareness and understanding of ourselves can improve the way we work, contributing to our personal development, but also to society as a whole.

In this article, we report on the outcomes of a 30‐credit master‐level (UK level 7) module that was specifically designed to develop and enhance employability skills of science students via a 6‐week placement at a UK organisation or overseas partner university. The purpose of this module is for the students to gain work‐based experiences that cannot be obtained during conventional projects in our host laboratories. Key to the learning outcomes of this module are as follows: (a) instructional guidance to the students on the expectations of the placement and their role in the experiential learning process and personal development; (b) self‐evaluation by students of their perceived competencies in a range of employability skills before, during and after the placement, coupled with self‐reflection of how these skills have been developed and can be demonstrated; and (c) awareness of the impact or beneficiaries of the work being undertaken and how this work can benefit society. External examiners and employers have commented very favourably on the outcomes of this module on students’ skills development and preparedness for the workplace. The aims of this study were (a) to determine the students’ perceptions of a proposed structured reflective process that supports the development of their transferable skills while on placement, (b) to explore students’ perceptions of the value of this module for their career development and employability prospects, and (c) to assess their awareness of the personal skills developed during a placement. As highlighted above, the purpose of the educational intervention was not to measure the difference between students’ skill levels pre‐ and postplacement, but to support and increase students’ awareness and perception of their own skills levels, and to offer an understanding of how well they are able to link these with their wider development as life scientists.

## Materials and methods

### The module and placements

The placement module is for students on the Integrated Masters of the School of Life Sciences at the University of Liverpool, UK. An Integrated Masters in the UK is a four‐year degree, where the first 3 years constitute the bachelor years, and the fourth year is the master year. The placement activity runs in the summer between years 3 and 4 of study and must be conducted at a location outside the School of Life Sciences. The placement accounts for 30 out of 120 credits of the fourth year. It is expected that this corresponds to 300 h of learning time, which includes learning time in the host institution of about 230 h. The remaining time is allocated for researching the placement topic before and after the placement, and the time spent on preparing for other module assessments, once students are back at the university (see next section for more detail).

There are many types of placement opportunities available to the students. They should select one that will give them the type of additional experience that will be important for their future career. Students have the opportunity to work abroad or in the UK. The idea is to experience a very different approach to research influenced by local factors (economic or social) that are pertinent to the region of the world in which they are working. For both types of placements, national and international, students should understand the economic or social factors influencing their work/research and should learn about why the particular work is being done and who is likely to benefit if the work is successful. During the placement, a staff member from the host institution supervises the student’s work. In addition, a University of Liverpool staff member provides the support needed for the student’s learning and personal development. This includes a combination of pastoral support and guidance during for students’ self‐evaluation of the perceived competencies developed in a range of employability skills (see next section for more detail).

To support students’ preparation for the placement, we run placement predeparture activities, where we discuss:

- The importance of engagement with the project literature: students are presented with the topic of their placement research and are instructed to contact their host supervisors for more information on the project;
- The importance of the work conducted: we stress some key differences between the student/university lifestyle and the professional settings, and cultural differences if appropriate;
- The role of the host and the university supervisors, and their expectations;
- The module overview: learning outcomes and assessments.

### Recording and evaluating skills

Based on the knowledge that bioscience students need more training and guidance in reflective practice [22] and students are assessment‐driven [23], the Online Reflective Log summative assessment was introduced to the module in the 2016–17 academic year and is completed before and during the 6‐week placement. It is weighted as 25% of the total module assessment, engaging and supporting students in reflecting on skills development [24]. The other two assessments, completed after students return to the university campus, are the placement report and an oral presentation, weighted as 65% and 10%, respectively. The Online Reflective Log aims to foster students’ self‐evaluation as students have to reflect on and record evidence of their acquired transferable skills. Students in the past tended to struggle when asked to reflect on their development and acquired skills. For this reason, their university supervisor offers feedback and guidance on the completion of the Online Reflective Log to students during their placement. In addition, the Log helps the university supervisors to monitor and identify if students encounter any problems during their placement; they are then able to support students while off‐campus. The Online Reflective Log has three main learning and teaching purposes: (a) formative feedback (guidance to the new placement experience encouraging students to evaluate their employability skills development); (b) summative assessment (provide a mark to the work as a way to boost engagement); and (c) diagnostic (a regular dialogue between university staff and students allows for identification of problems).

**Fig. 1:**
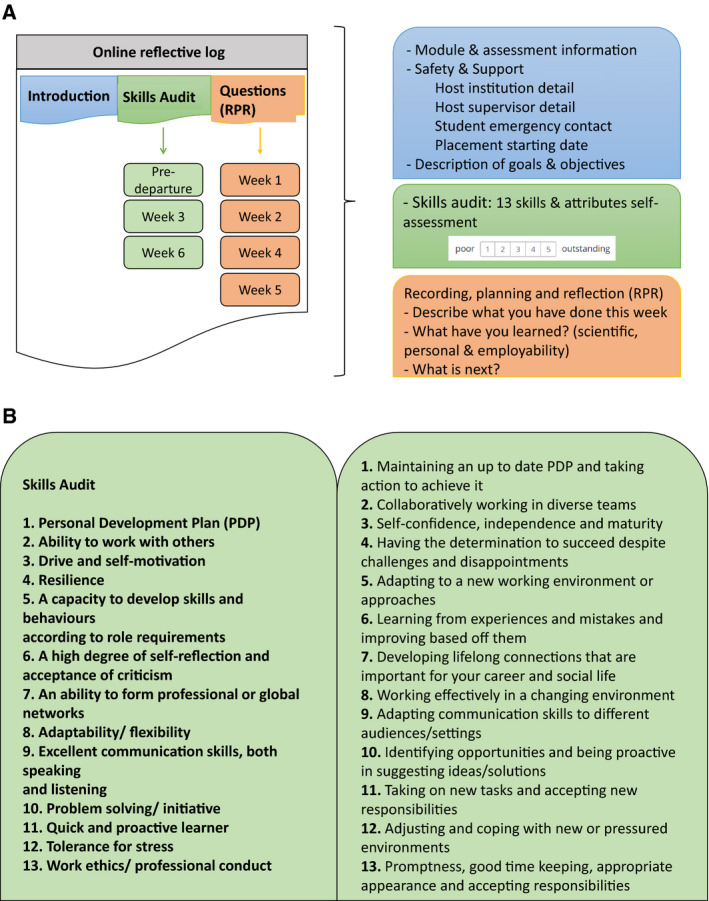
Online Reflective Log: (A) The three components: introduction, skills audit and questions. The time of completion: predeparture and last day of the indicated week in the boxes; (B) skills audit: students score themselves (corresponding to predeparture, week 3 and 6) and give evidence on 13 main attributes and employability skills (week 6).

Students should engage with the Online Reflective Log prior to starting the placement, and then every week during the placement. The Log is divided into three main parts as shown in Fig. 1A,B. The first, the *Introduction & Goals* part, focuses on providing information on the module learning outcomes and assessments. In addition, it asks the students to provide information about the placement host institution, and the reasons for taking the placement – its goals and objectives. It is worth noting that these are not in relation to the placement research but rather what employability skills can be developed during this placement experience. The second part is the Skills Audit that consists of 13 skills and attributes. Students are asked to perform a self‐assessment using a scale of 5 (1 poor–5 outstanding) (Fig. 1B). These scores are not intended to provide quantitative measurements, but instead to prompt students to reflect on their own skills and identify areas for improvement. This reflective exercise is done three times, predeparture and in weeks 3 and 6. In week 6, the last week of the placement, students are also asked to explain the scores given on the three occasions, providing evidence (250 words per skill attribute) for any change, or no change, in the score, reflecting on their personal skills and development. The third part is the RPR (Recording, Planning and Reflection). This consists of a brief description of the work done in the week, and two questions: (a) What the student learned/gained from the work done in the week (scientific and employability skills) and (b) the plans for the next week. Students complete this weekly activity in the Online Reflective Log by the end of every placement week.

To support students with completing the Online Reflective Log, we also discuss the following topics in the placement predeparture activities:

- The Online Reflective Log: How to use the online activity platform, the log structure (Fig. 1) and deadline for weekly task completion, and how to complete each part of the log (examples are shown to the students);
- Employers expectations and employability skills: students are provided with literature on skills development and the benefits of completing a skills audit;
- The skills audit: we explain that the emphasis of this activity is on the reflection and evidence of skills development, or lack of a skills (students are not evaluated on the scores themselves);
- The dynamic between students and university supervisors: students are assigned to a particular supervisor who provides feedback to the students. Students are asked to complete the weekly log between Friday and Monday, after a week of work. Formative feedback is provided by Tuesday in weeks 2, 3 and 5, and summative feedback (and assessment) is provided after the completion of the placement. The Online Reflective Log is a structured and scaffolded reflective process that is supported by the supervisor and their feedback as an ongoing dialogue throughout the placement.

In summary, the six weekly online forms contain specific elements for students to complete, which are then read and evaluated online by University of Liverpool supervisors who provide timely feedback and suggestions for improvement. This log also includes a skills audit, highlighting both the scientific and personal skills that students have developed during their placement. Monitoring students’ ongoing performance is important in order to encourage formative feedback and dialogue between students and the university supervisors, as well as encouraging students to take responsibility for their learning.

### Ethics and research questions

This work was funded by the School of Life Sciences, University of Liverpool. Ethics approval was granted by the University of Liverpool’s Ethics Committee in March 2016. Informed consent was sought and granted by participants. This study’s research questions were as follows: (a) What are students’ perceptions of the value of this placement experience for their career development and employability prospects? (b) How do the students recognise the skills developed as part of their placement? and (c) What was the impact of the Online Reflective Log on students’ awareness of their skills development?

Punch [25] describes empirical research as research based on direct experience or observation of the world involving two main kinds of data, quantitative or qualitative. Since the research questions are framed in an interpretative paradigm, research methods focusing on qualitative data were chosen as the most suitable. Quantitative data collection was used to prompt student reflection about awareness of skills development with respect to changes (e.g. increase or decrease). A questionnaire and a focus group were used to collect student feedback after the introduction of the Online Reflective Log to the module. In addition, students were invited to comment on the module in a school‐wide end‐of‐module evaluation. We adopted a sequential study, using the focus group to identify common themes and areas to explore in the subsequent surveys [26].

The structured focus group was facilitated to enable students to freely discuss the online resources, and encouraging them to think about their placement experience and how it was evaluated. This then enabled us to develop the questionnaire to address the study’s research questions. Free‐text comments from questionnaires and the focus group transcript were analysed using thematic analysis [27]. All participants were fourth‐year students in the School of Life Sciences. Students were provided with information sheets prior to their participation, and participants signed consent forms for the focus group.

### Focus Group

A focus group is a way of conducting a collective interview, using an unstructured, semi‐structured or structured approach [26]. The focus group took place with eight fourth‐year students from the School of Life Sciences in 2016–17, facilitated by one of the authors (TVA). An email was sent out to all fourth‐year students inviting them to participate in a focus group, which explored themes arising from a pilot survey that students had previously completed about their placement experience (not shown here). Participants were selected as they took part in an elective 6‐week summer placement, abroad or in the UK. The host sites included research laboratories, research support offices, clinical trial units and hospital settings. The 60‐min session was recorded and transcribed. The session was structured exploring the following themes: placement experiences; impact of the placement; employability skills acquired during the placement; and skills development enhanced by the Online Reflective Log exercise/assessment. The focus group findings were fed into the design of a new questionnaire to evaluate this study. For consistency, the same questionnaire design was then used to compare all years of study.

### Questionnaires

Questionnaire‐based research is a well‐known and commonly used research approach in educational research [28]. This study used two different questionnaires to collect students’ views: the study questionnaire and the module evaluation survey used for evaluating modules in the department. Questionnaire data were collected for three consecutive academic years: 2017/18, 2018/19 and 2019/20. The number of students on the module for these academic years was *N* = 38, *N* = 23 and *N* = 30, respectively. Students completed both questionnaires voluntarily and anonymously after all the coursework and assessments.

The study questionnaire, designed based on the focus group analysis, contained ten questions; two consisted of Likert‐scale data exploring students’ perception of the impact of the placement on their skill development. Three were open questions asking the students to list the most and least beneficial aspects of taking a placement, and the impact of a placement on their future career plans. Two questions addressed the Online Reflective Log assessments as learning opportunities. The remaining three questions were as follows: a demographic one, the students’ master specialisation subject and suggestions for improvement of the placement provision. In addition to this questionnaire, we analysed data collected via the module evaluation survey in all 3 years of study. This is a standard questionnaire for all modules, designed to gather students’ feedback on the module, including questions on content, teaching approach and assessments. For this study, we focus on the open questions of the evaluation questionnaire, where students are asked to list up to three particular strengths of the module.

## Results

**Fig. 2:**
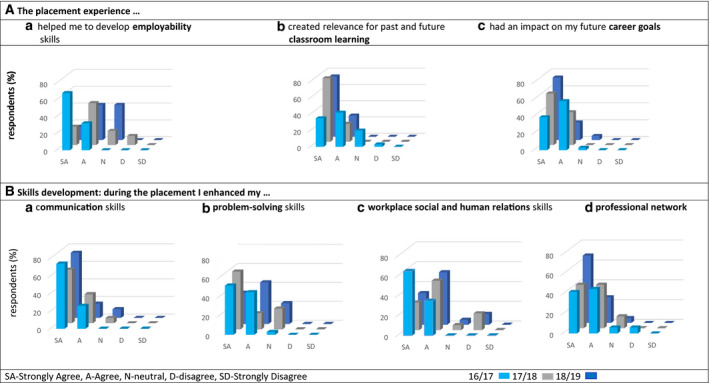
Percentage of respondents agreeing/disagreeing with statements regarding the placement. In (A), statements relate to the placement experience, and in (B), statements relate to skills development during the placement. Cohort size and % of respondent for each year of analysis: 2017/18, *N* = 38, respondents = 82%; 2018/19, *N* = 23, respondents = 78%; and 2019/20, *N* = 30, respondents = 74%.

**Fig. 3:**
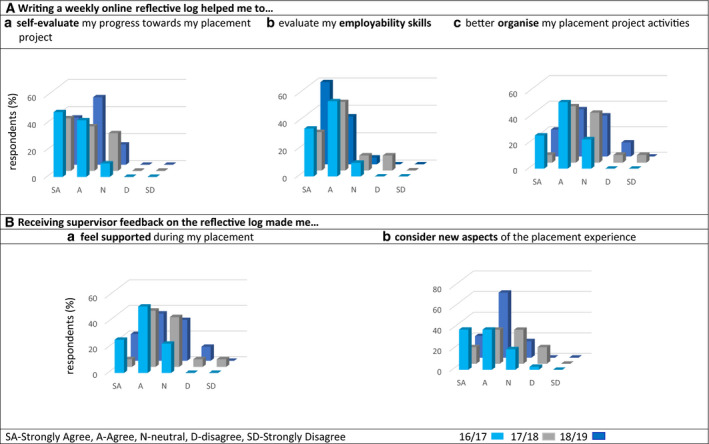
Percentage of respondents agreeing/disagreeing with the statements regarding the Online Reflective Log. In (A), statements relate to the weekly completion of the weekly online report, and in (B), statements relate to the university supervisor on the reflective log during the placement. Cohort size and % of respondent for each year of analysis: 2017/18, *N* = 38, respondents = 82%; 2018/19, *N* = 23, respondents = 78%; and 2019/20, *N* = 30, respondents = 74%.

The quantitative and qualitative results were analysed in three main categories according to the aims of the study: students’ perception of (a) the impact of the Online Reflective Log on their skill development; (b) the value students placed on their placement experience for their career development and employability prospects; and (c) students’ perception on the skills developed as part of their placement. Quantitative questionnaire data are mentioned as three values, corresponding to the three academic years of data collection. The response rates of the study questionnaire were 82%, 78% and 74% in the academic years of 2017/18, 2018/19 and 2019/20, respectively. Figures 2 and 3 show that students almost always strongly agree or agree that the placement experience benefited their skills development and that the introduction of the Online Reflective Log assessment to the module was essential to support the reflective exercise requested from the students in this module.

### The placement experience

Students were positive about their placement experience. Figure 2A shows that 84%, 72% and 58% of the students either strongly agree or agree that the placement experience helped them to develop employability skills; 77%, 100% and 100% either strongly agree or agree that the placement experience created relevance for past and future classroom learning; and 97%, 100% and 95% either strongly agree or agree that the placement experience had an impact on their future career goals. These results were supported by the qualitative data of the module evaluation survey that illustrates the most common benefits of the module mentioned by the students (Table 1). When compared to the focus group results, the findings reinforce the two themes that emerged with respect to the overall impact of the placement: linking the laboratory/work day by day with personal experiences, and expanding or confirming career aspirations. A number of students commented that, as a result of taking part in the placement and conducting their own independent research at the host site, they were better able to make connections between their scientific knowledge, usually constrained within the walls of the undergraduate science laboratory, and the wider impact of their research on society and people. One student commented that in the laboratory as a student they felt ‘detached from any sort of benefit to people!’, while during the placement they saw professionals in action, helping them to draw the links between science in the laboratory and science as it impacts on people and society. Students related this realisation with the Online Reflective Log exercise, where they were constantly asked to reflect and discuss the purpose of their research project in line with the module aims (see results and analysis below).

**Table 1:** Themes and typical students’ comments on employability skills development during the placement.

Theme	Typical quotes
Professional and life skills development awareness	*Very good at developing professional skills in a working environment* *Gained such good life experience and developed my skills such as independence* *Learned on how to reflect on what I have learned* *The reflective scenario was critical in developing and appreciating the new employability skills that were acquired*
Multicultural and linguistic skills (abroad placements)	*Learned about a different culture* *I challenged myself to learn another language. I ended helping my colleagues in daily and professional situations Amazing!* *Experienced the globalised nature of research*
Work in a new environment	*Adapting really quickly to a new environment there* *I remember thinking I have got these skills…but the new environment make me realise I wasn’t* *as good as I thought I was* *The clinical experience allowed me to understand about patients consent and confidentiality* *Exposed us to new research environment where it is being effectively being translated to treat patients*
Independence as a researcher	*Not just undertaking research tasks, but designing it and with the supervisor taking more of a back seat (as opposed to UG projects)* *I had a set research question and had to make a timetable to fit everything in*
Being aware and managing tasks within the wider team members’ tasks	*So it was like a lot of things on top of each other that I had managed rather than just my own research*
Professionalism and maturity	*I think the placement allows you to behave more in a professional manner because it allows the transition when you actually go out there. You know how to behave professionally; you know what people are looking for, what the employers are looking for. But they want you to be mature in every way possible and this placement provided that opportunity to develop*

Expanding on career choices and aspirations, the focus group showed that some students already had an idea of what setting they wanted to work in (e.g. pure research or clinical). However, they claimed that the placement experience had either confirmed this prior choice or opened new horizons. They were also able to appreciate a wider range of research settings (e.g. research‐support or mix of clinical and laboratory research), of which they were previously not aware. In one case, the placement helped student re‐assess their previous perceptions of certain research‐related jobs, having earlier dismissed them as ‘office jobs’, but now seeing them as something more exciting and desirable. Others realised that they would like to have more links with the human/patient side of the research and started considering a clinical setting for their future career. This diverse response reveals the benefit of offering different types of placement, not only the most common industrial placements.

### The skills developed during the placement

Students were extremely positive about their development of the four skills described in Fig. 2B with responses of either ‘strongly agree’ or ‘agree’ of 100%, 94% and 90% for communication skills; 97%, 78% and 77% for problem‐solving; 100%, 78% and 85% for social and human relations; and 88%, 88% and 95% for professional networking development. It is worth noting that for the academic year 2019/20, this result contradicts those from Fig. 2A(a), where a high number of students said the placement experience was neutral with respect to the development of their employability skills. A similar positive response was seen in the quantitative analysis of the final‐year module evaluation, where students saw the link between communication skills, human relations and networking (Table 1). In the focus group, students expressed their appreciation of being in a new setting for their research experience, as they appreciated the learning process in relation to networking with colleagues: ‘*I would say I learnt the most from actually talking to the researchers and sort of just working within the team’*. Table 1 shows further examples from the focus group discussion of students developing employability skills as a result of the placement. One of the students commented that although they were actively looking through job applications during their degree and learning what employers are looking for via the job specification, it was only through the placement that he was able to understand what these skills meant. Many students also mentioned this realisation in the final‐year module evaluation, linked to the importance of the Online Reflective Log: *‘the placement taught new skills and the reflective scenario imposed by the reflective log was critical in developing and appreciating the new employability skills that were acquired’*.

### The impact of the Online Reflective Log on students’ skill development

The Online Reflective Log consisted of three main parts: introduction, skills audit and questions. The introduction part was used for information about the student and the placement. This study analyses students’ responses to the last two parts, the skills audit and questions (Fig. 1).

Figure 3A shows the student’s evaluation of the benefits of the Online Reflective Log prior to and during their placements. Once again, students almost always ‘strongly agreed’ or ‘agreed’ with the statements: 90%, 72% and 85% for self‐evaluation of students’ progress towards their placement project; 90%, 78% and 95% for evaluation of their employability skills; and 81%, 83% and 84% for organisation of their placement project activities. However, for this last category, a higher proportion of students chose ‘neither’, indicating a less beneficial impact of the Online Reflective Log for this particular activity. As previously mentioned, one of the aims of the Online Reflective Log was to improve support to the students while off‐campus by identifying their development needs. When asked about the benefits of receiving regular feedback from their academic supervisor via the Online Reflective Log, 87%, 76% and 82% of the respondents either ‘strongly agreed’ or ‘agreed’ that they felt supported during their placements (Fig. 3B). In addition, 78%, 49% and 84% revealed that the feedback made them consider aspects of the placement experience that they had not thought about before their interaction with the University of Liverpool supervisor (Fig. 3B). The Online Reflective Log was praised as an excellent learning tool, regularly mentioned in the final module evaluation survey and focus group. A student commented that *‘The weekly Online Reflective Log was great for keeping students motivated and forced students to think about the cultural and employability aspects’*. While another student said that the Online Reflective Log *‘challenges you to reflect on your personal development’*. The comments from the focus group were grouped by themes and are presented in Table 2.

**Table 2:** Students’ comments on the use of the Online Reflective Log during the focus group.

(a) The weekly Online Reflective Log helped you remember: *It was really useful to have the reflective work that we’d done every week because then you could just look and see what you’ve been doing and just add it all together and decide. So it was all there so you haven’t forgotten anything like you might have if you weren’t* *logging it down every week* *It is only for 6 weeks: timeframe awareness*
(b) The impact of taking notes: *It is the writing down process that makes you realise what you have developed, or picked something up* *When you write it down you realise you’ve actually picked up something and that’s one of the skills the employability skills that the employers look for*
(c) Reflection: *I am not a reflective person* *– the skill to reflect didn’t* *come naturally to us, it was something that we had to learn, which in turn helped us to be able to self‐evaluate our actions* *Reflection does not come naturally and I have never asked to reflect on my development, at this extent, before*
(d) Progression and development – whether as scientists, researchers or professionals: *As the 6‐weeks passed, looking back through the logs, we could see that we could do more work in the time allotted* *The frequency of this task made us more aware of their timeframe. It encouraged us to do more with your time* … *critique yourself as well* *The audit skills is a good opportunity to evaluate yourself*
(e) Use of literature in employability: A particularly useful aspect of the Online Reflective Log was that students were encouraged to consult the literature on what employers were asking; this helped them be more aware of what skills they were developing: *It’s not necessarily picking up the skills but looking through the literature which they kept encouraging us to do. It did actually make you realise some things that employers might be looking for that you weren’t* *aware of. Like one of the things that I found was quite a large percentage wanted employees to be open to working in different environments and maybe across the globe and that experience of working abroad like made me want to work in different countries throughout my career*

**Fig. 4:**
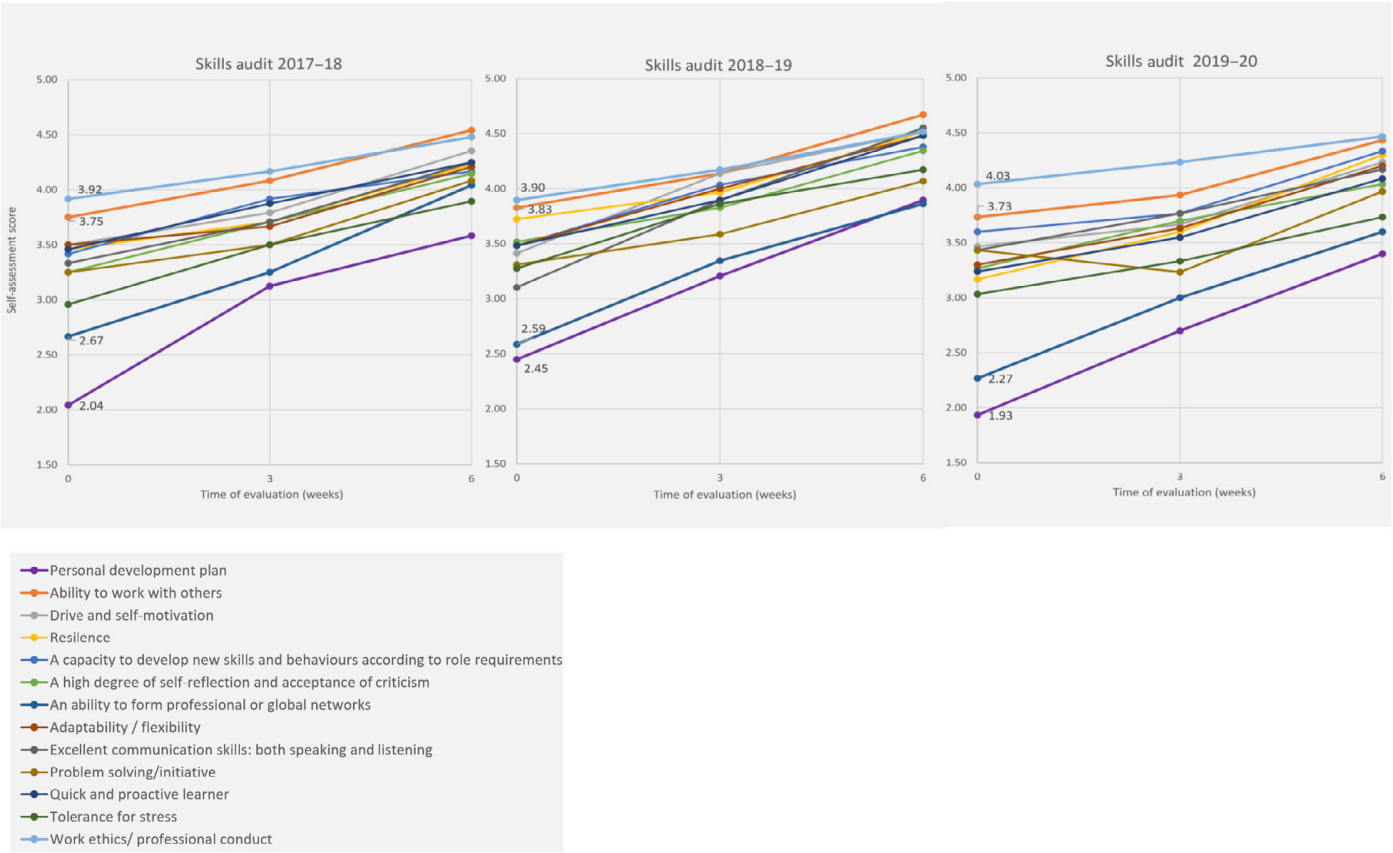
Self‐assessment skills audit average scores for week 0 (predeparture), week 3 and 6 (time of evaluation). The values shown on the graphs are the predeparture average scores for the fours skills discussed in the text: personal development plan (2.04), ability to work with others (3.75), an ability to form professional or global networks (2.67) and work ethics/ professional conduct. Cohort size for each year of analysis: 2017/18, *N* = 38, 2018/19, *N* = 23, and 2019/20, *N* = 30.

Figure 4 shows the average skills audit scores per year of study. These are students’ self‐evaluations at three points: week 0 (predeparture), week 3 and 6. Among many scores presented in the figure, two were consistently higher before the placement – *work ethics/ professional conduct* (light blue), and *ability to work with others* (orange), and two were consistently low before the placement – *personal development plan* (purple), *an ability to form professional or global networks* (dark blue). The data pose some questions on the students’ perception of their readiness, after 3 years of study, for planning their next steps and networking. When designing curricula, we should provide students with either more opportunities to develop those skills or, if they are already on the curricula, with the opportunity to recognise and reflect on them. While we could speculate that Fig. 4 shows an overall increase in students’ perception of skills development (also shown in Fig. 2 and Table 1), the data should be interpreted with caution. Although the students used a quantitative scale (1–5) to rate their skills, the purpose of this was to prompt student reflection and awareness of changes in skills development (e.g. increase or decrease) to prompt self‐reflection of their skills and to identify areas for improvement. Therefore, despite an overall perception of skills gain in the summary data, a close analysis of the data showed that for some individual students, their scores remain unchanged or even dropped over the placement, because they overestimated their competence in a particular skill before the placement began. Other students may have deliberately underscored their skills in the first audit so that they could show improvement by the end of the placement. It is worth noting that students were assured that in this module, they would be assessed based on the quality of their reflection and narrative evidence provided, rather than the scores themselves. For these reasons, we did not further analyse these skills audit scores. Some students identified and commented on such fluctuating trends (higher–lower–higher scores) for their self‐evaluation of skills preplacement–during–postplacement, for instance: ‘*This sounds really arrogant but I remember thinking I’ve got some of these skills and I think I’m going to be quite good at this and then by week three, because you’ve been up half the time and you’ve got used to it, you realise that maybe in that environment because it was new you weren’t* *as good as you thought you were … so that brought my scores back down and towards the end the other thing was coming to a close and starting to get all the results, it brought my scores back up because I was like I’ve managed to achieve all of this and I’ve done all of these things that maybe week three me would’ve struggled with. So I think that’s why mine [came] back up in the end*’. Another student highlighted the helpfulness of being supported to audit and reflect on their skills via the weekly logs: ‘*I actually thought it was really helpful doing the audit because it did let me put into context what I’d been doing and the fact that my academic supervisor asked us to write reference papers and that meant I had to go out and read things. So I did find it quite helpful but I started out thinking my professionalism, my time management is great and then midway through you’re okay it could be better like when you compare it to people in a professional setting rather than in my honours project setting*’.

As the intended outcome of the reflective process was for students to recognise and evidence their skills, such fluctuation in reported skill levels was expected by university supervisors. However, as the placement progressed, decreasing scores were perceived as indicative of failure by some students. This showed that a clearer explanation of the aims of this exercise is needed, namely to increase awareness of personal strengths and weaknesses, followed by a plan for further development of each skill. This observation led to changes in the module, and from the academic year 2018–19, more support for skills audit completion was offered to the students, prior departing to their placements.

## Discussion and Conclusion

The research questions of this study were to determine the following: (a) What are students’ perceptions of the value of this placement experience for their career development and employability prospects? (b) How do the students recognise the skills developed as part of their placement? and (c) What was the impact of the Online Reflective Log on students’ awareness of their skills development? To answer these questions, we gathered students’ views using a focus group and, from the themes revealed, we subsequently developed a survey to explore students’ perceptions of their skills development. We repeated this for three consecutive years, minimising any cohort bias.

Our results show that the placements helped students to develop both scientific and transferable skills. One may argue this is a well‐known outcome. However, this study shows skills development during a 6‐week placement, instead of the more conventional year‐long in industry placements. Furthermore, the reflective process structured by the Online Reflective Log assessment was key to raising students’ awareness of their strengths and weaknesses, supporting lifelong learning. The benefits of using reflective logs to support students’ development of employability skills have already been established [29]. More recently, Hill and collaborators [30] showed that science students reported ‘unfamiliarity of thinking beyond knowledge attainment in order to identify and reflect on skill‐related experiences’. Their study also showed that students recognised that by improving their ability to reflect, they would be better prepared to articulate their skills when seeking employment. The need to improve students’ reflective ability was also illustrated by Voelkel *et al*. [22], where it was shown that students’ willingness and ability to reflect on their learning process during their final‐year dissertation were patchy, indicating that more training in the skills of reflection earlier on in their course was needed.

Building on these findings, we can say that one of the main contributions of this paper to the literature on placements and skills development is the focus on the ongoing formative feedback and summative assessment of skills development, including students’ self‐reflection and recording of their development, as part of a credit‐bearing module. Placement students tend only to be assessed through a report on their placement work and experiments, that is on their scientific results. In this study, however, we encouraged the students to reflect on the process by which they gained and developed employability skills, and to use this reflective process to design their own personal development plans. While other studies have reported on the use of skills audits to help students’ self‐evaluation of acquired skills in response to a particular activity, the reflective component has not been explored [2, 31]. Kensington‐Miller and collaborators [32] presented a framework to help undergraduates recognise, articulate and evidence attributes or skills that are often developed or required within a university degree and profession, but are not explicitly discussed or assessed. In this module, students have to explain the changes in their skills audit scores, for example they need to provide evidence on how skills were developed, or seemed to be lacking: *‘it is the writing down process that makes you realise what you have developed, or picked something up’* (student).

Beard [31] claims that ‘*the value of a placement experience is enhanced when the experience is systematically assessed*’. The results of our study showed the benefit of reflective activity embedded in a credit‐bearing module to foster skills development. The Online Reflective Log helped students to critically assess their skills and keep a permanent record of performance and development. We argue that recording skills may serve as a launchpad for self‐reflection and continuing professional development. Studies show that employers often look for ‘soft’ skills as critical for professional success alongside traditional ‘hard’ skills [11, 33]. Thus, it could be argued that the structured reflective process as part of the Online Reflective Log should better prepare the students for future job applications and interviews, increasing their employment chances, whether in industry, in clinical settings or in academia.

The science curriculum should allow for more reflection and employability skill development. A common trend observed by supervisors when providing feedback on students’ Online Reflective Log tasks during their placement (RPR section) was that in the first 2 weeks, students mainly focused on reporting their weekly scientific laboratory experiments: What was done and plans for the following week. The feedback from the university supervisors was essential to bring additional transferable skills development experiences and planning into students’ discussions. This demonstrates that skills development reflection does not come naturally to students studying science. The reflective process should go beyond an organised and detailed laboratory book: it should go beyond scientific skills. Therefore, we emphasise the importance of the reflection process being well‐structured and supported by university supervisors. This means paying attention to the following: (a) How often do students evaluate their skills development and (b) How do we help students with skills recording and reflection.

Another aspect to consider is that in general, placements either are offered in HEIs as extra‐curricular activities, or have a low weight towards a student degree; for example a year in industry may count for only 10% of the overall degree mark. Other studies present ideas of how to develop extra‐curricular activities to develop students’ employability skills (e.g. [2]). The module described here is worth a total of 25% of the students’ master year total credits (30 out of 120 credits), demonstrating our emphasis on skills development in the curriculum. In addition, the Online Reflective Log here proposed was used as a summative assessment weighted 25% of the module. The summative aspect was used to ensure students’ engagement with the reflective activity. This module has developed further in the current academic year, 2020–21. We are now working in collaboration with the university’s Careers and Employability Centre who supplement the placement predeparture activities by delivering three talks: What employers look for in graduates; what you can gain from placements; and how to complete a reflective log. The benefit of the Online Reflective Log on students’ learning can be demonstrated by its incorporation into three other modules in the School of Life Sciences. Students are now asked to complete a skill audit in years 1, 2 and 3, and reflect on their development during their years of study. The Online Reflective Log has been adopted by other schools/departments at our university, and its benefits for supporting students off‐campus can be illustrated by a quote from a colleague: *The strategic Online Reflective Log monitoring system is a powerful tool to support students on placement. It allows us to check on the students’ wellbeing while off‐campus, and guide employability skills development*. Outside our institution, the Online Reflective Log with the structured support by academic supervisors has been implemented as part of other placement activities/modules: a colleague at a different university reported that they adopted the Online Reflective Log in the 2019–20 academic year. Furthermore, they noted that students were writing reflective diary style notes in the margin of the laboratory books as their project progressed. This demonstrates the importance of establishing a structured process of supporting students towards being able to recognise the transferable skills they are developing as part of their degree course bridging the gap between the classroom and the workplace.

Another unique aspect of this study was to focus on the outcomes of a 6‐week science placement instead of a year‐long one. Students’ responses demonstrated evidence that skills development can be achieved during a shorter placement. In addition, the placements were offered to students in several settings, not just in industry, as students’ careers aspirations vary. The results illustrate that different working environments provide the students with common skills development, while they can also develop students’ appreciation of different challenges, for example cultural and language barriers, and the different priorities in industrial, clinical and academic settings [34].

In summary, the module was well received by the students and has allowed them to develop key employability skills within a shorter, 6‐week period. The Online Reflective Log has emphasised students’ initial difficulties with the reflection process, but has also demonstrated that if properly supported and supervised, students will engage with the reflection. There is no doubt that a placement experience fosters skills development, but a well‐organised and carefully structured, supervised and assessed experience can help students to achieve a better understanding and awareness of their skills development. Placement assessments should not only focus on the final scientific outcome but also focus on the whole process of personal and scientific development.

## Conflict of interest

The authors declare no conflict of interest.

## Author contributions

LVM conceived the study, and LVM and SWE designed the project. LVM and TVA acquired the study, and analysed and interpreted the data. LVM and SWE wrote the manuscript. TVA commented on multiple versions of the manuscript.

## Data Availability

All data not subject to limited distribution due to confidentiality agreements are available upon reasonable request to the first author, LVM.
